# Supplementary material for: synNotch-programmed iPSC-derived NK cells usurp TIGIT and CD73 activities for glioblastoma therapy
Source: Nat Commun. 2024 Mar 1;15:1909. doi: 10.1038/s41467-024-46343-3 (PMC10907695; doi:10.1038/s41467-024-46343-3)
Supplement: Supplementary file 1 — Supplementary Information [file 41467_2024_46343_MOESM1_ESM.pdf]

Supplementary Information
for

synNotch-programmed iPSC-derived NK cells usurp TIGIT and CD73 activities for glioblastoma therapy

Kyle B. Lupo,[1] Xue Yao,[1] Shambhavi Borde,[1] Jiao Wang,[1] Sandra Torregrosa-Allen,[2] Bennett D. Elzey,[2,3] Sagar Utturkar,[2] Nadia A. Lanman,[2,3] MacKenzie McIntosh,[4] Sandro Matosevic[1,2,*]

[1]Department of Industrial and Molecular Pharmaceutics, Purdue University, West Lafayette, IN, USA

[2]Center for Cancer Research, Purdue University, West Lafayette, IN, USA

[3]Department of Comparative Pathobiology, Purdue University, West Lafayette, IN, USA

[4]Histology Research Laboratory, Center for Comparative Translational Research, College of Veterinary Medicine, Purdue University, West Lafayette, IN, USA.

*Corresponding author. Email: sandro@purdue.edu

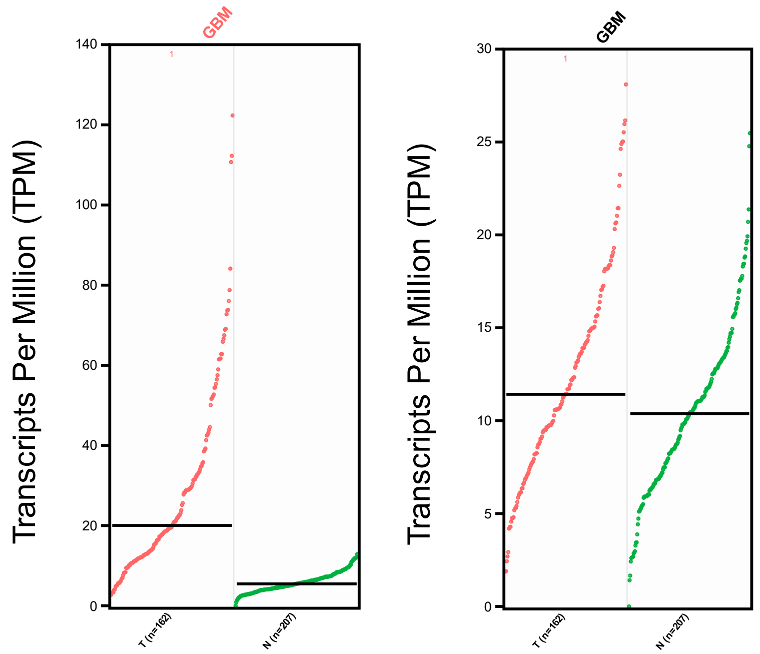

Supplementary Figure 1. TCGA data quantifying transcripts per million (TMP) of *NT5E* (CD73; *left*) and *PVR* (CD155; *right*) on GBM tumor (n=162 patients) (*red*) and normal (n=207 patients) (*green*) tissue.

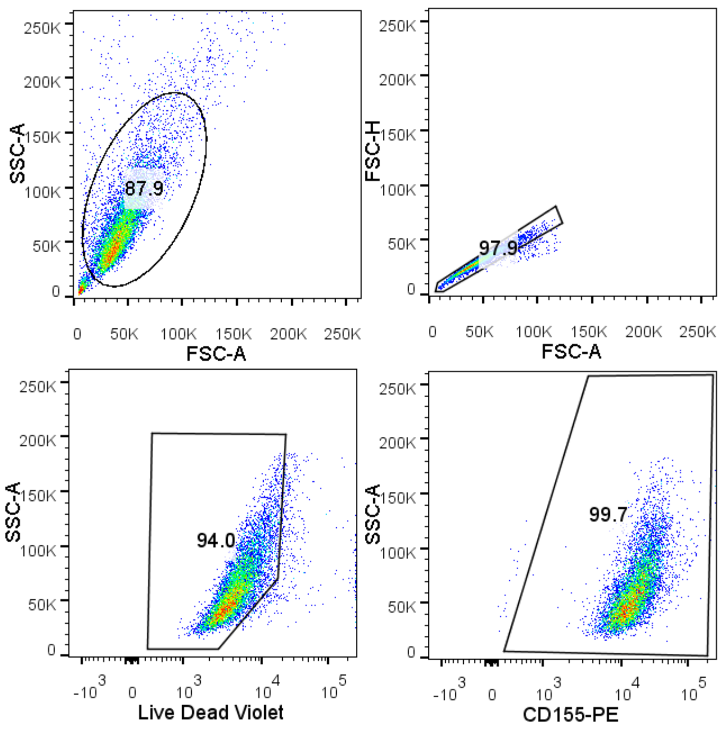

Supplementary Figure 2. CD155/CD73 flow cytometry gating strategy on GBM target cells (*Figure 1I, Supplementary Figure 3*).

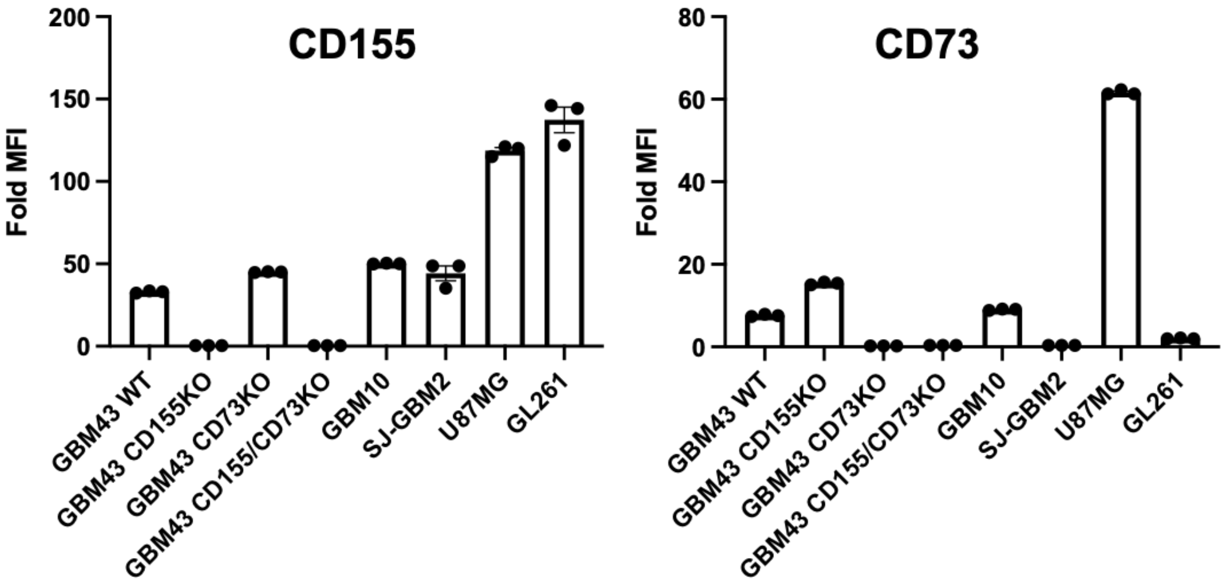

Supplementary Figure 3. Fold change of MFI over isotype control of CD155 and CD73 expression on GBM target cells (n=3 independent experiments). Data are presented as mean values +/- SD. Source data are provided as a Source Data file.

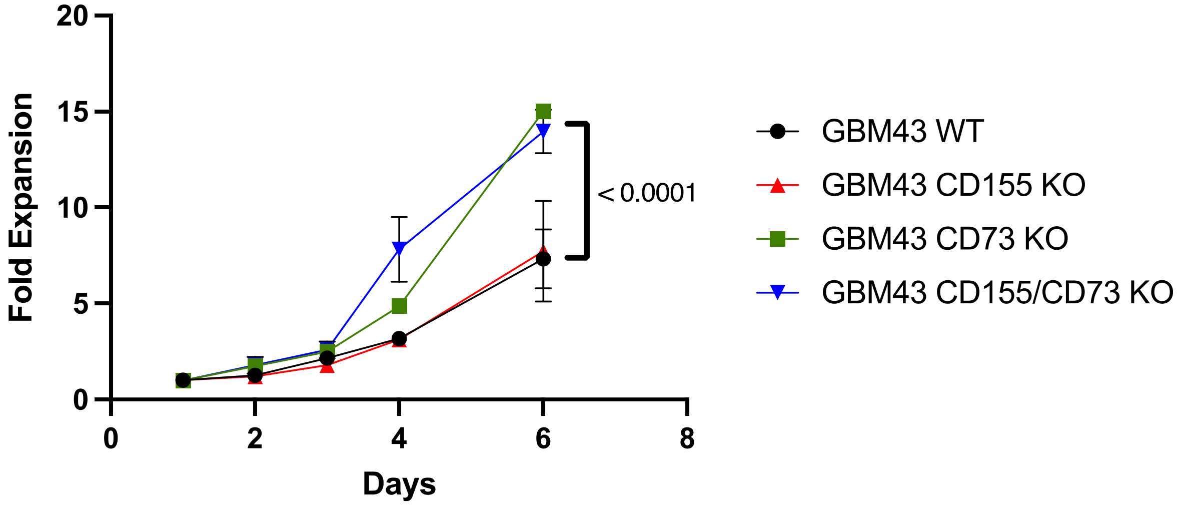

Supplementary Figure 4. Proliferation of GBM43 WT and CRISPR/Cas9 KO cell lines measured by CCK-8 (n=3 independent experiments; two-way ANOVA, Tukey's multiple comparison test). Data are presented as mean values +/- SEM. Source data are provided as a Source Data file.

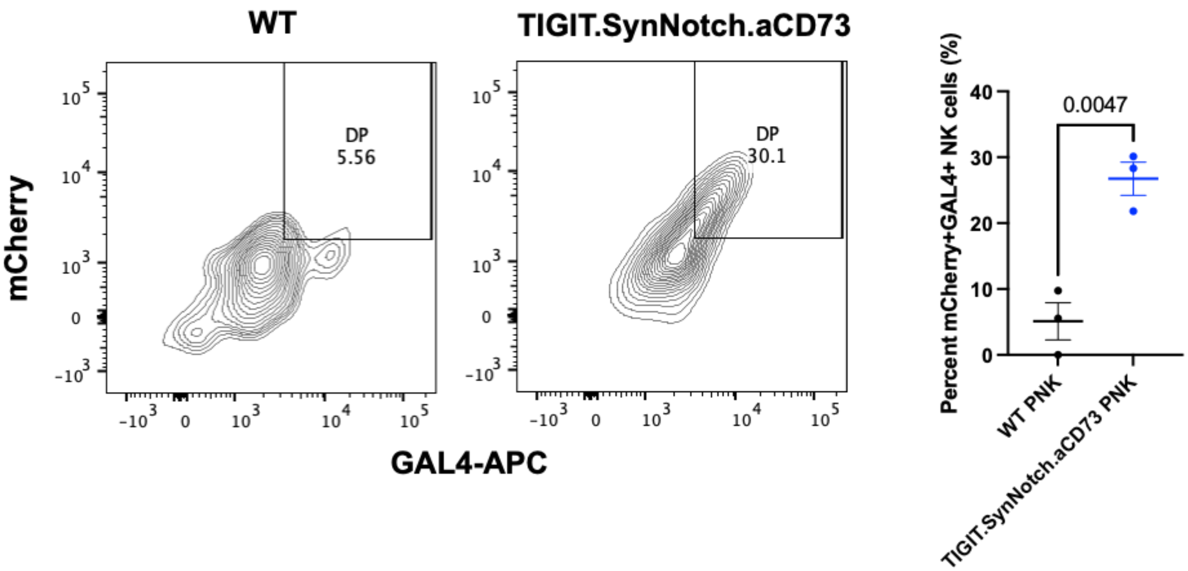

Supplementary Figure 5. GAL4 and mCherry expression of WT and TIGIT.SynNotch.aCD73 primary NK cells measured via flow cytometry (n=3 independent experiments; unpaired two-tailed t-test). Data are presented as mean values +/- SEM. Source data are provided as a Source Data file.

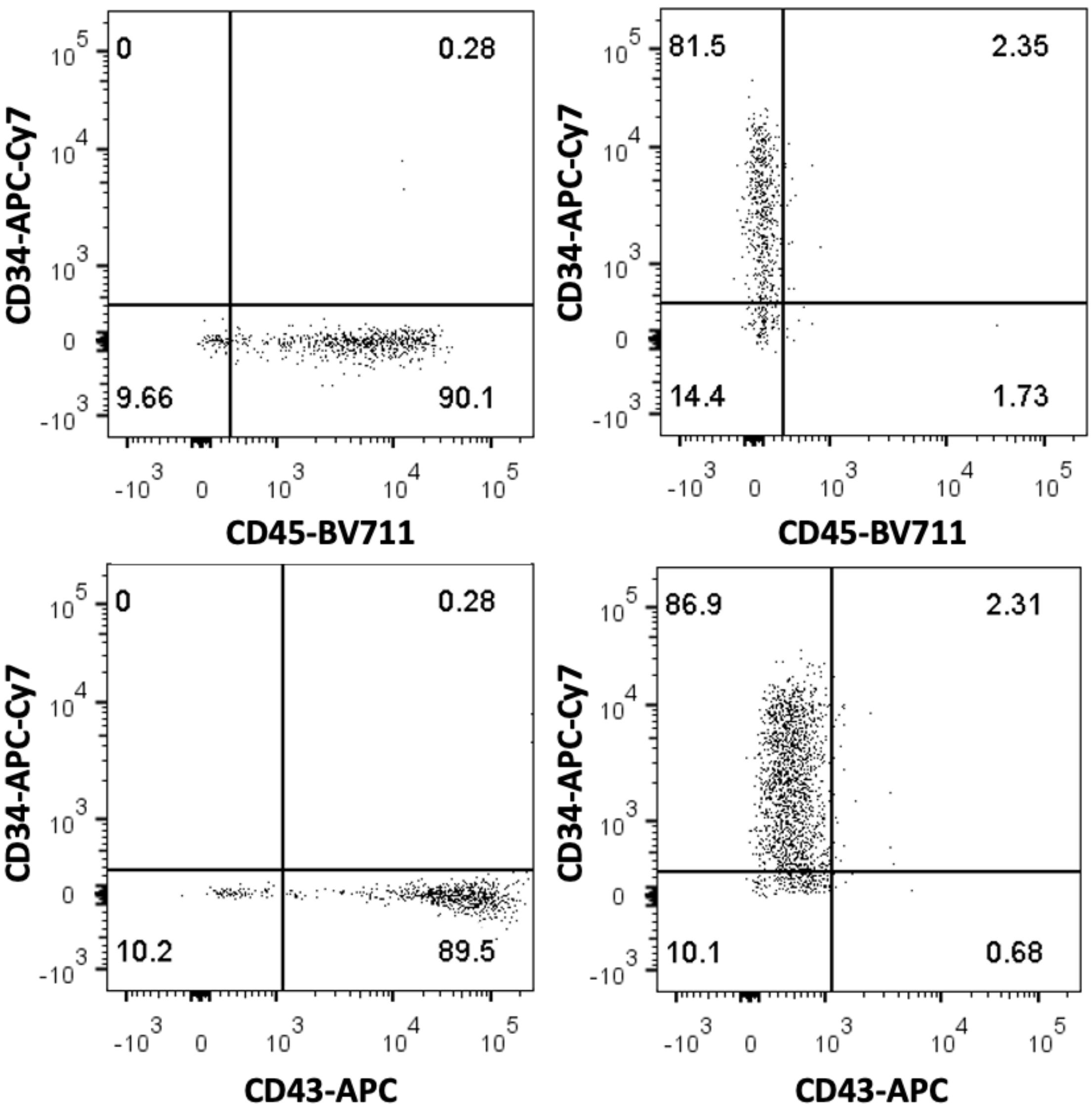

Supplementary Figure 6. Flow cytometry dot plots depicting isotype controls for CD34, CD43, and CD45 on iPS-derived hematopoietic progenitor cells (*Figure 2E, 2F*).

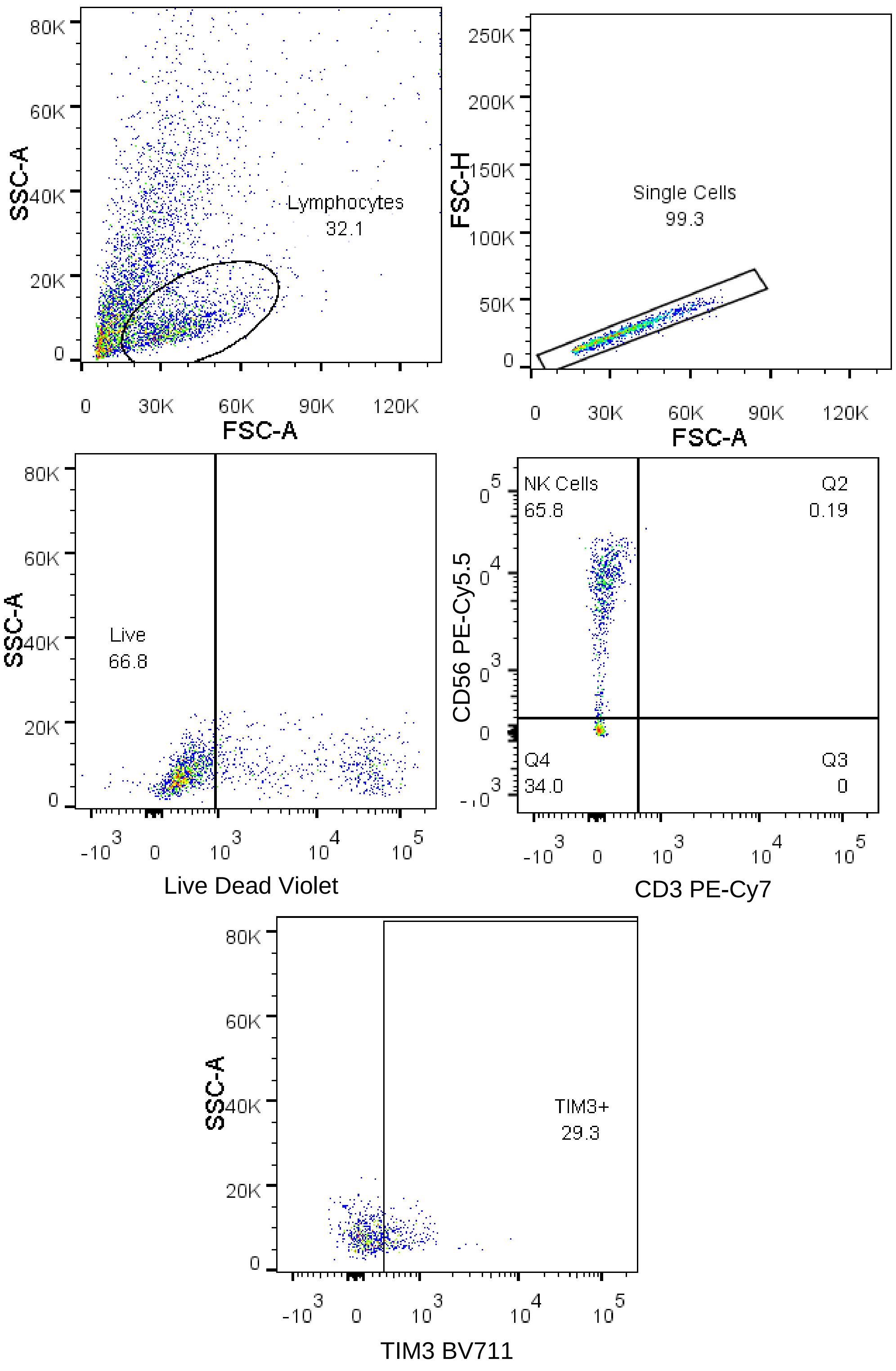

Supplementary Figure 7. Flow cytometry gating strategy for staining of iNK cells *in vitro* and *in vivo (Figure 2C, 2D, 2G-L, 3B, 3J-L, 6K-L).*

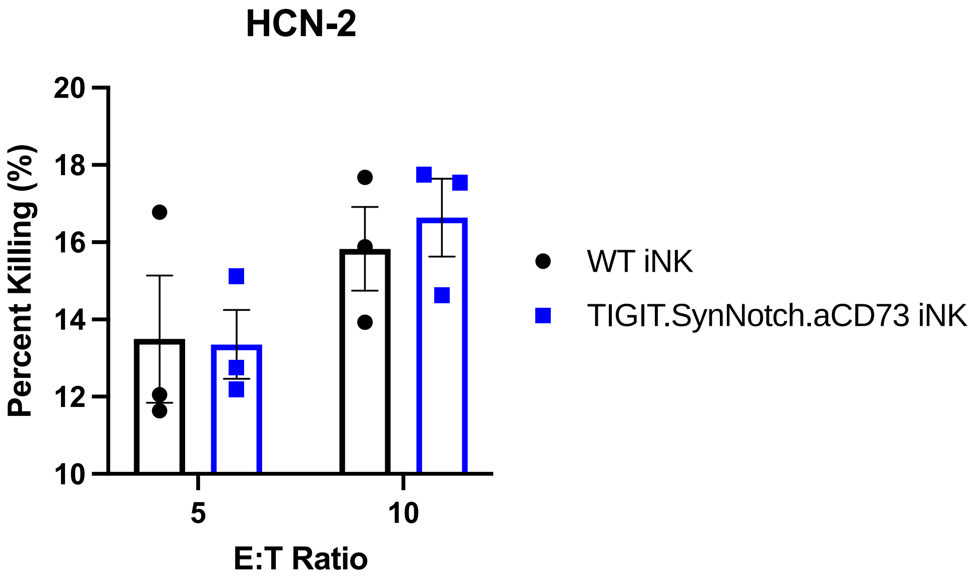

Supplementary Figure 8. Cytotoxicity of engineered iNK cells versus WT iNK against normal cortical neuron cell line HCN-2 (n=3 independent experiments; multiple unpaired two-tailed t-tests). Data are presented as mean values +/- SEM. Source data are provided as a Source Data file.

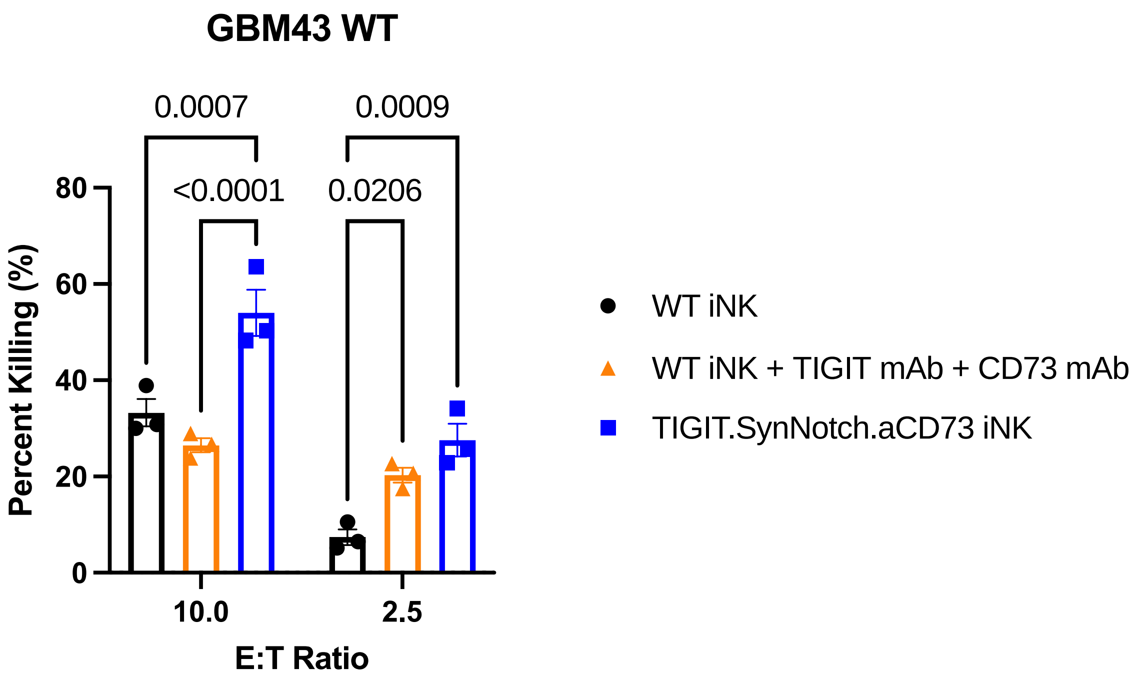

Supplementary Figure 9. Cytotoxicity of TIGIT.SynNotch.aCD73 primary NK cells versus WT primary NK cells with or without mAb blockade of TIGIT and CD73 against GBM43-WT primary patient derived GBM target cells (n=3 independent experiments; two-way ANOVA, Tukey's multiple comparison test, simple row effects). Data are presented as mean values +/- SEM. Source data are provided as a Source Data file.

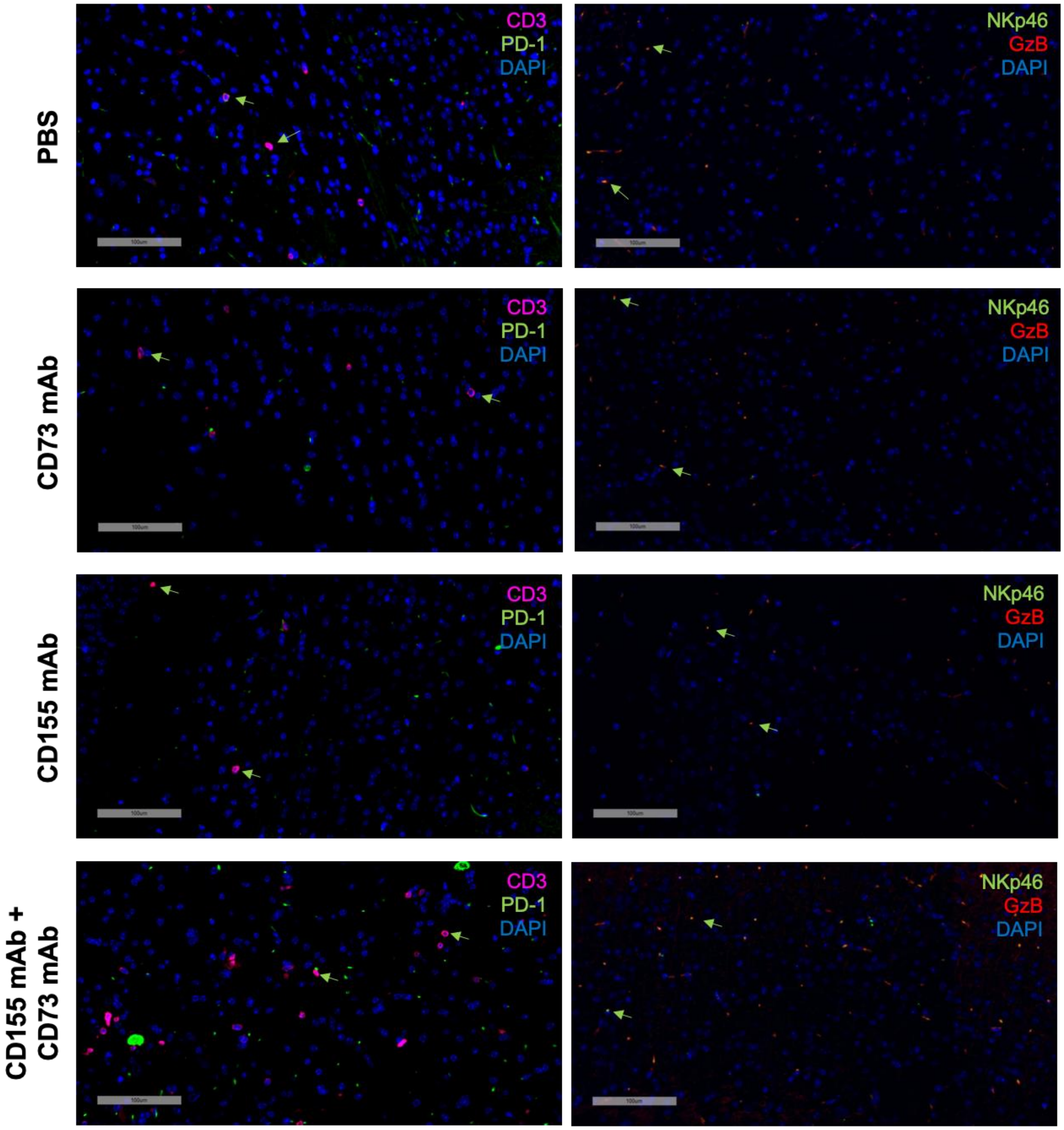

Supplementary Figure 10. Immunofluorescent staining of CD3 (*red*) and PD-1 (*green*), and NKp46 (*green*) and GzB (*red*) on tumor sections for intracranial GL261 tumor-bearing mice following treatment with PBS, CD155 mAb, CD73 mAb or both CD155 and CD73 mAbs; GzB = Granzyme B.

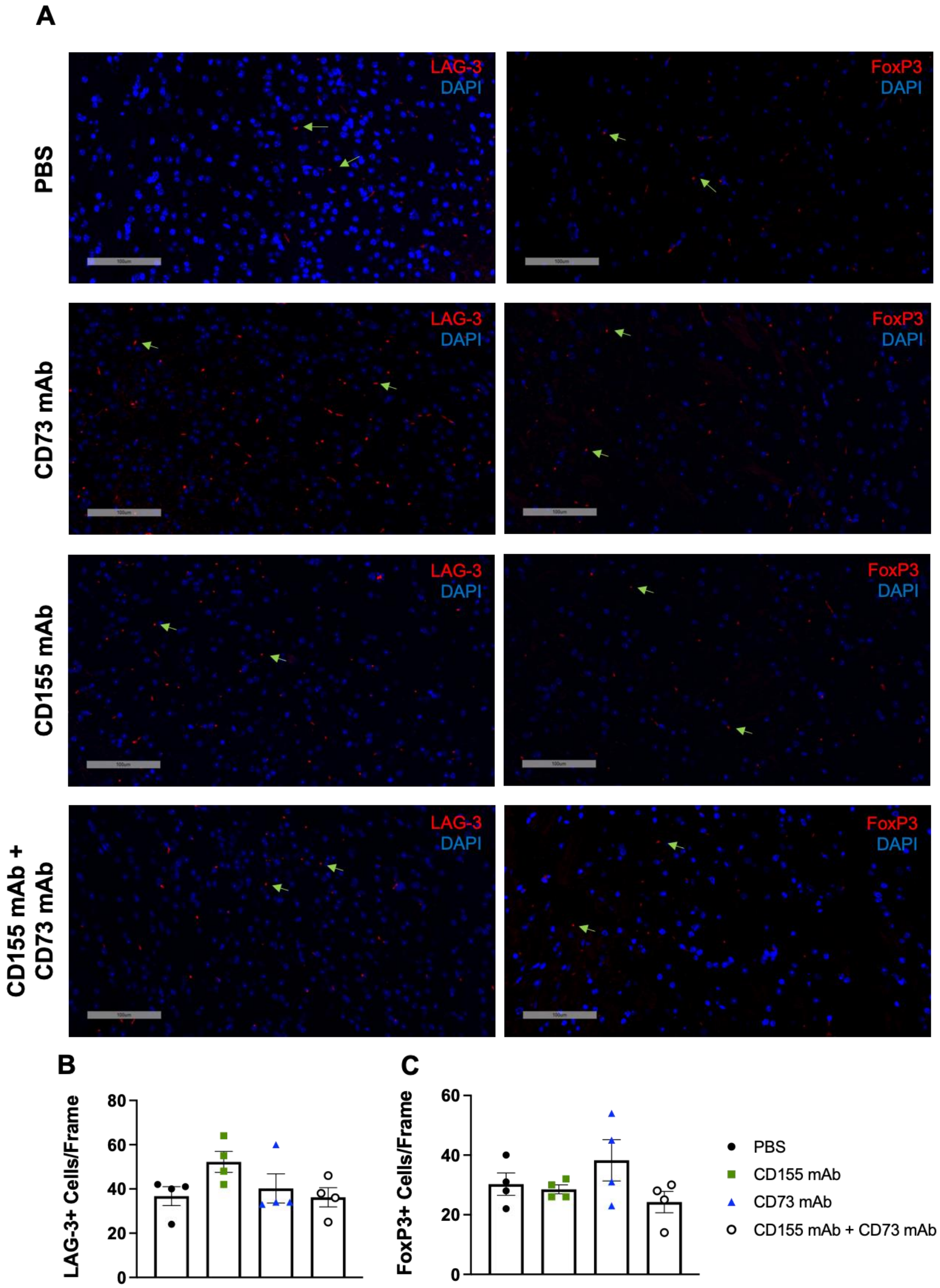

Supplementary Figure 11. (A) Immunofluorescent staining of LAG-3 and FoxP3 on tumor sections for intracranial GL261 tumor-bearing mice following treatment with PBS, CD155 mAb, CD73 mAb or both CD155 and CD73 mAbs. (B-C) Quantification of immunofluorescent staining depicted in *Supplementary Figure 11A* (n=4 intratumoral sections; ordinary one-way ANOVA, Tukey's multiple comparison test). Data are presented as mean values +/- SEM. Source data are provided as a Source Data file.

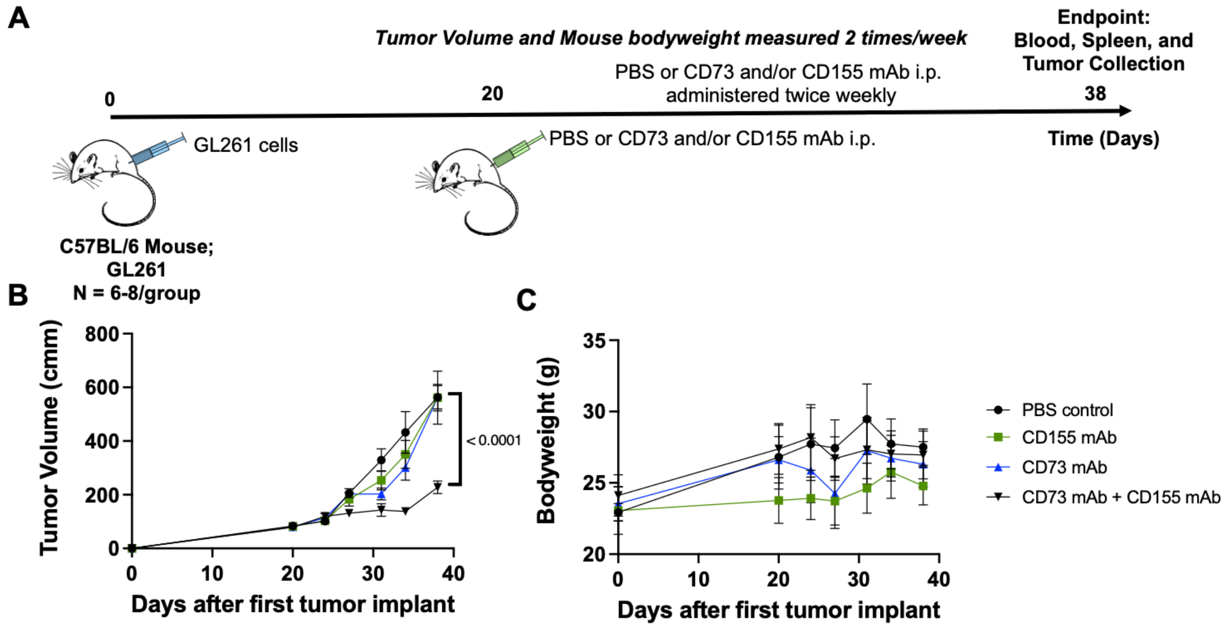

Supplementary Figure 12. (A) Study timeline of GL261 flank tumor-bearing mice treated with or without CD73 and/or CD155 mAb; PBS - n=8 mice, CD155 mAb - n=6 mice, CD73 mAb - n=6 mice, CD73 mAb + CD155 mAb - n=8 mice. (B-C) Tumor volume and bodyweight measurements of mice measured throughout the course of the study (Two-way ANOVA, Tukey's multiple comparison test). Data are presented as mean values +/- SEM. Source data are provided as a Source Data file.

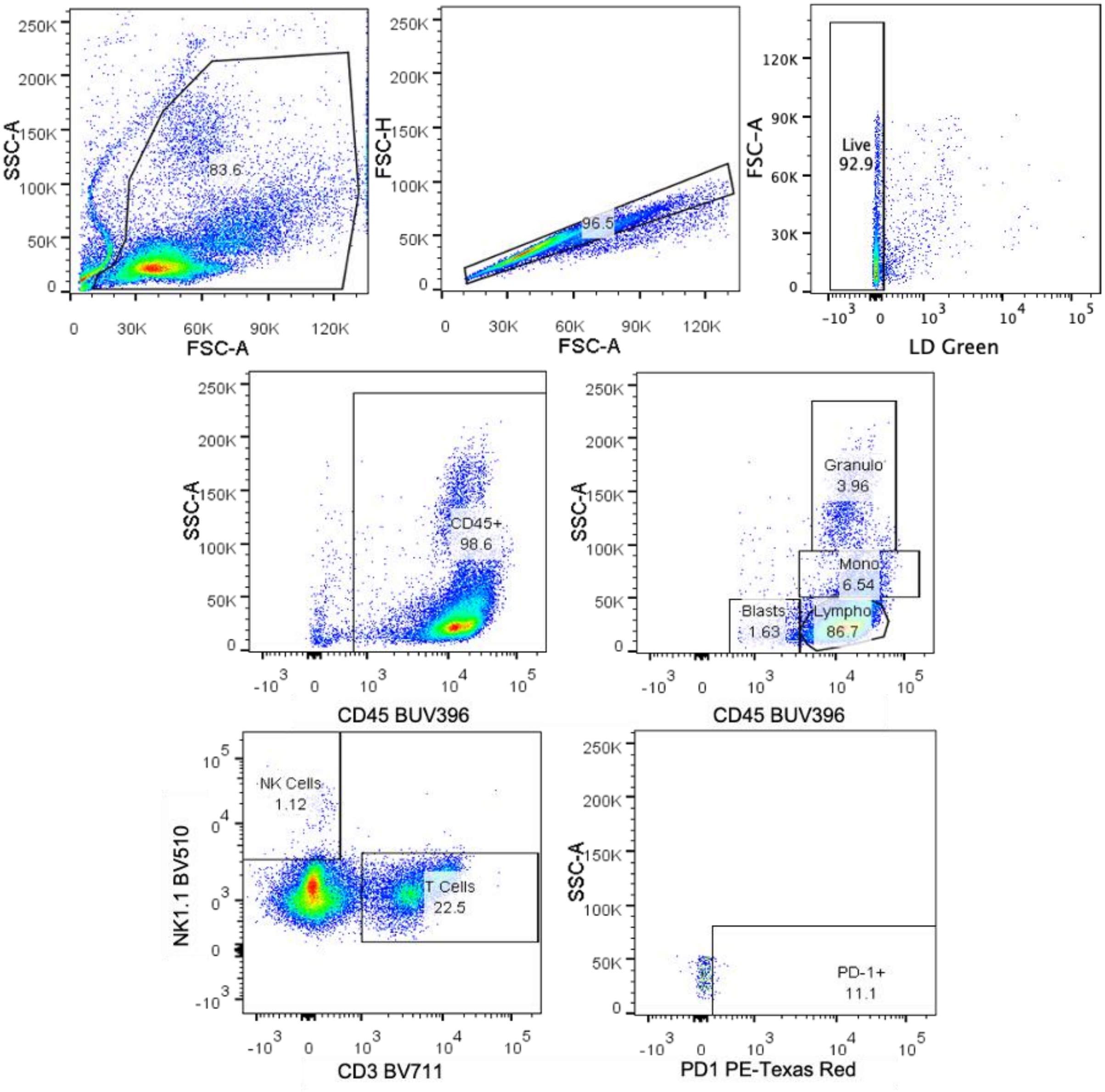

Supplementary Figure 13. Flow cytometry gating strategy for staining of mouse immune cell types (*Figure 4H-I, Supplementary Figures 14-15*).

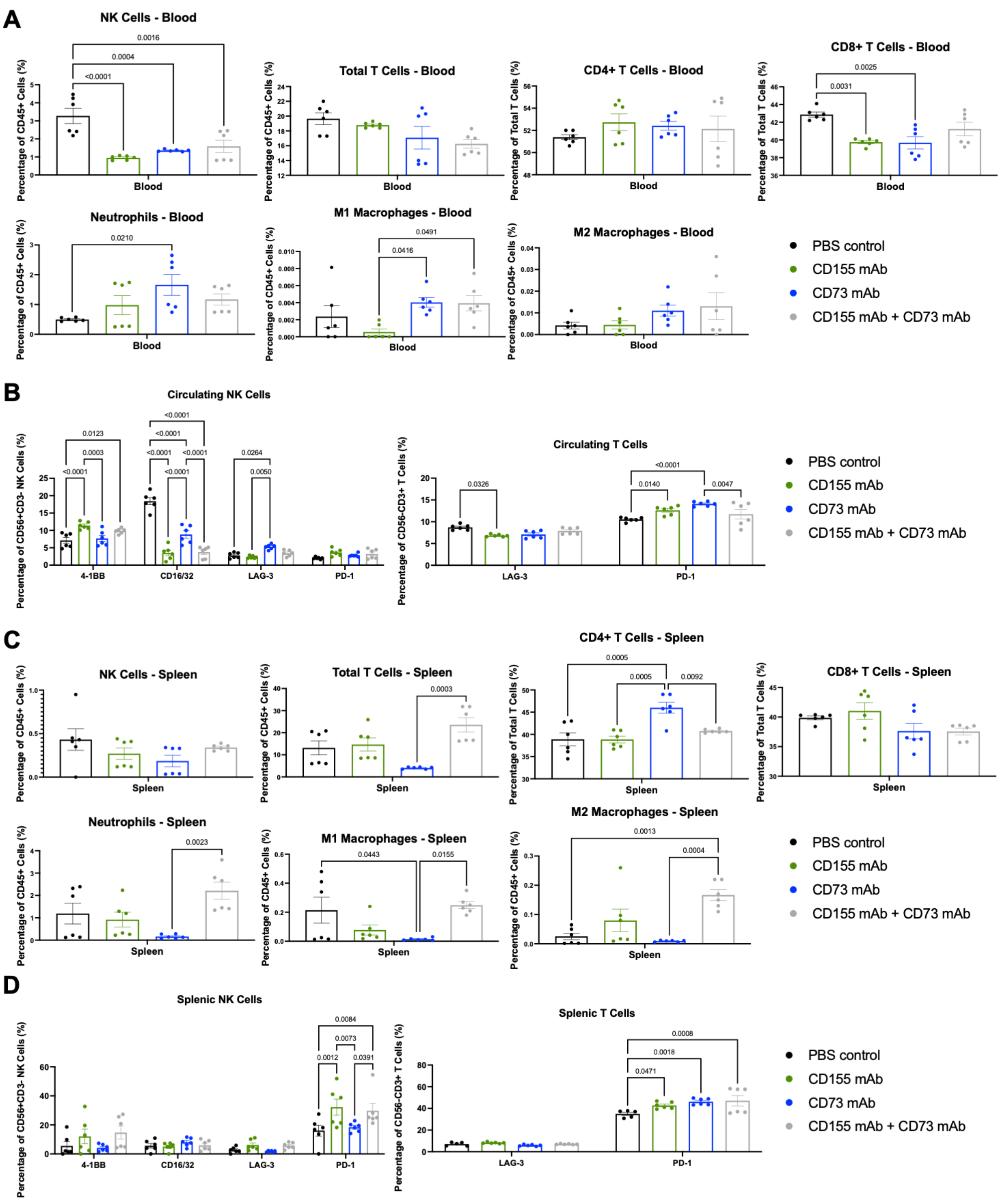

A
NK Cells - Blood
0.0016
0.0004
<0.0001
Percentage of CD45+ Cells (%)
Blood
Total T Cells - Blood
Percentage of CD45+ Cells (%)
Blood
CD4+ T Cells - Blood
Percentage of Total T Cells (%)
Blood
CD8+ T Cells - Blood
0.0025
0.0031
Percentage of Total T Cells (%)
Blood
Neutrophils - Blood
0.0210
Percentage of CD45+ Cells (%)
Blood
M1 Macrophages - Blood
0.0491
0.0416
Percentage of CD45+ Cells (%)
Blood
M2 Macrophages - Blood
Percentage of CD45+ Cells (%)
Blood
PBS control
CD155 mAb
CD73 mAb
CD155 mAb + CD73 mAb
B
Circulating NK Cells
Percentage of CD56+CD3- NK Cells (%)
4-1BB
CD16/32
LAG-3
PD-1
Circulating T Cells
Percentage of CD56-CD3+ T Cells (%)
LAG-3
PD-1
PBS control
CD155 mAb
CD73 mAb
CD155 mAb + CD73 mAb
C
NK Cells - Spleen
Percentage of CD45+ Cells (%)
Spleen
Total T Cells - Spleen
0.0003
Percentage of CD45+ Cells (%)
Spleen
CD4+ T Cells - Spleen
0.0005
0.0005
0.0092
Percentage of Total T Cells (%)
Spleen
CD8+ T Cells - Spleen
Percentage of Total T Cells (%)
Spleen
Neutrophils - Spleen
0.0023
Percentage of CD45+ Cells (%)
Spleen
M1 Macrophages - Spleen
0.0443
0.0155
Percentage of CD45+ Cells (%)
Spleen
M2 Macrophages - Spleen
0.0013
0.0004
Percentage of CD45+ Cells (%)
Spleen
PBS control
CD155 mAb
CD73 mAb
CD155 mAb + CD73 mAb
D
Splenic NK Cells
0.0084
0.0073
0.0012
0.0391
Percentage of CD56+CD3- NK Cells (%)
4-1BB
CD16/32
LAG-3
PD-1
Splenic T Cells
0.0008
0.0018
0.0471
Percentage of CD56-CD3+ T Cells (%)
LAG-3
PD-1
PBS control
CD155 mAb
CD73 mAb
CD155 mAb + CD73 mAb

Supplementary Figure 14. (A) Percentage of various immune cells measured on circulating CD45+ cells in flank tumor-bearing immunocompetent mice measured via flow cytometry (Ordinary one-way ANOVA, Tukey's multiple comparison test). (B) Phenotypic analysis of circulating NK and T cells isolated from treated mice measured by flow cytometry (Two-way ANOVA, Tukey's multiple comparison test, simple row effects). (C) Percentage of various immune cells measured on splenic CD45+ cells in immunocompetent mice measured via flow cytometry (Ordinary one-way ANOVA, Tukey's multiple comparison test). (D) Phenotypic analysis of splenic NK and T cells isolated from treated mice measured by flow cytometry (Two-way ANOVA, Tukey's multiple comparison test, simple row effects); n=6 mice/group. Data are presented as mean values +/- SEM. Source data are provided as a Source Data file.

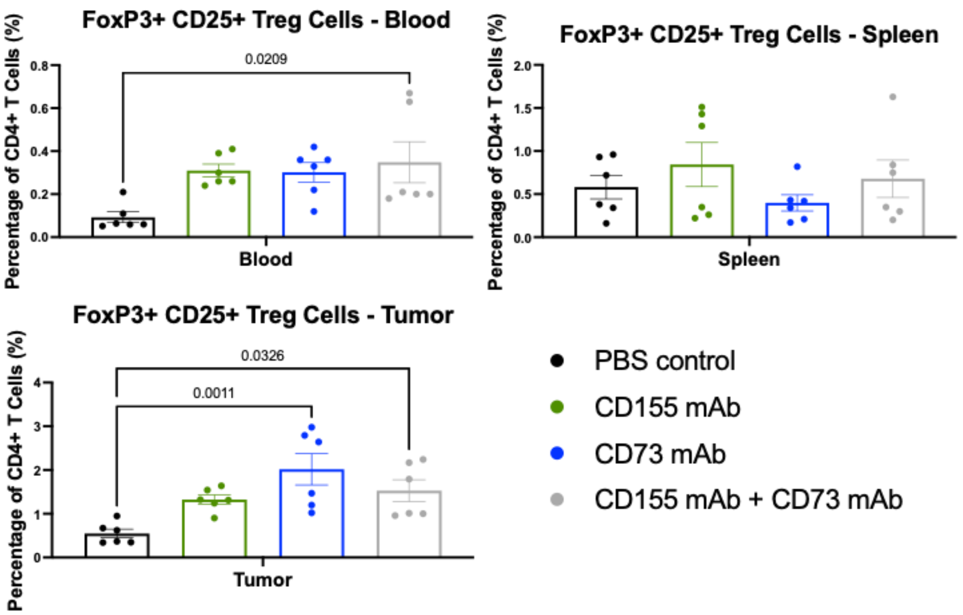

Supplementary Figure 15. Percentage of FoxP3+ CD25+ regulatory T cells (Tregs) gated on CD45+ cells in blood, spleen, and tumor tissue of flank tumor-bearing immunocompetent mice measured by flow cytometry; n=6 mice/group (Ordinary one-way ANOVA, Tukey's multiple comparison test). Data are presented as mean values +/- SEM. Source data are provided as a Source Data file.

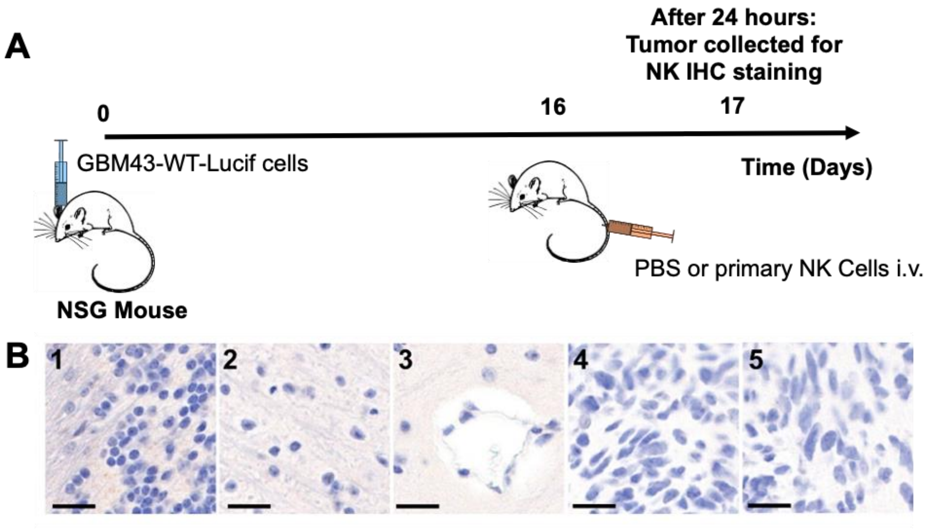

Supplementary Figure 16. Evaluation of NK cell homing following i.v. injection in an orthotopic patient-derived GBM xenograft model. (A) Schematic diagram showing the *in vivo* treatment program of GBM43 homing study. (B) Representative images of IHC staining for NKp46+ NK cells performed on brain sections from primary NK cell treated mice. Among them, (1-3) stand for normal brain tissue areas. (4-5) stand for tumor areas; n=5 mice Scale bar = 25 µm; 200× magnification.

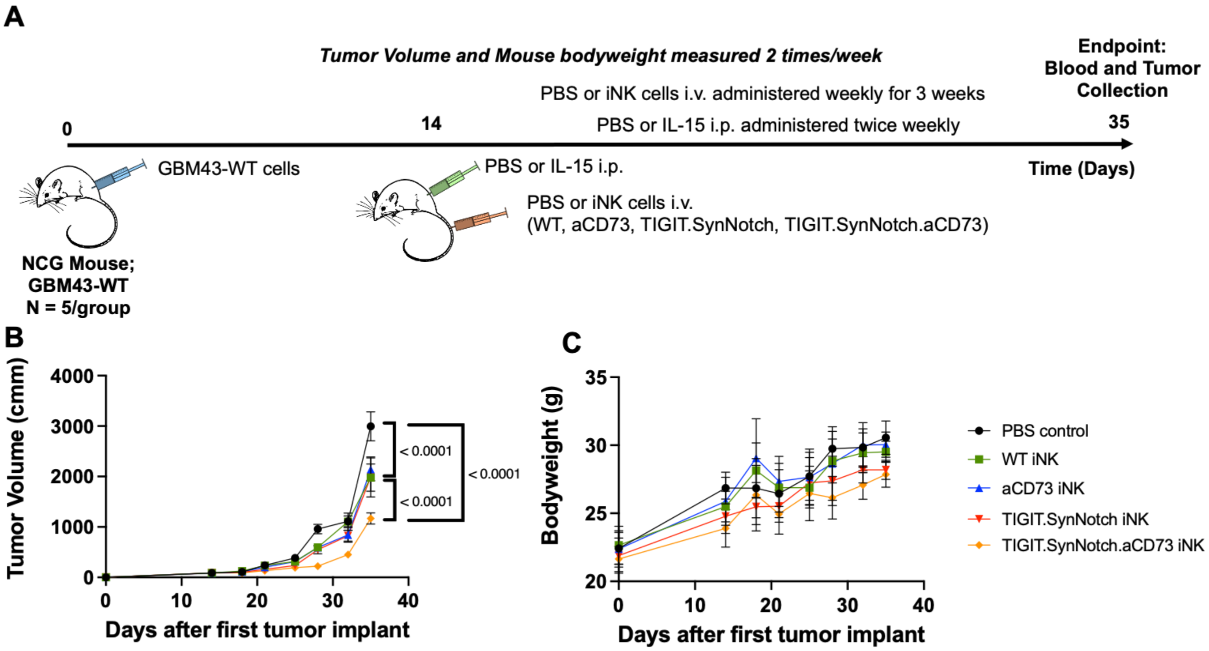

Supplementary Figure 17. (A) Study timeline of GBM43 WT flank tumor-bearing mice treated with or without non-engineered iNK cells or iNK cells engineered to express either a TIGIT.synNotch, aCD73, or TIGIT.synNotch.aCD73 genetic construct; n=5 mice/group (B-C) Tumor volume and bodyweight measurements of mice measured throughout the course of the study (Two-way ANOVA, Tukey's multiple comparison test). Data are presented as mean values +/- SEM. Source data are provided as a Source Data file.
