# Supplementary material for: synNotch-programmed iPSC-derived NK cells usurp TIGIT and CD73 activities for glioblastoma therapy
Source: Nat Commun. 2024 Mar 1;15:1909. doi: 10.1038/s41467-024-46343-3 (PMC10907695; doi:10.1038/s41467-024-46343-3)
Supplement: Supplementary file 2 — Reporting Summary [file 41467_2024_46343_MOESM2_ESM.pdf]

nature portfolio

Corresponding author(s): Sandro Matosevic

Last updated by author(s): Feb 20, 2024

Reporting Summary

Nature Portfolio wishes to improve the reproducibility of the work that we publish. This form provides structure for consistency and transparency in reporting. For further information on Nature Portfolio policies, see our Editorial Policies and the Editorial Policy Checklist.

Statistics

For all statistical analyses, confirm that the following items are present in the figure legend, table legend, main text, or Methods section.

n/a	Confirmed	
☐	☒	The exact sample size (*n*) for each experimental group/condition, given as a discrete number and unit of measurement
☐	☒	A statement on whether measurements were taken from distinct samples or whether the same sample was measured repeatedly
☐	☒	The statistical test(s) used AND whether they are one- or two-sided<br>*Only common tests should be described solely by name; describe more complex techniques in the Methods section.*
☐	☒	A description of all covariates tested
☐	☒	A description of any assumptions or corrections, such as tests of normality and adjustment for multiple comparisons
☐	☒	A full description of the statistical parameters including central tendency (e.g. means) or other basic estimates (e.g. regression coefficient) AND variation (e.g. standard deviation) or associated estimates of uncertainty (e.g. confidence intervals)
☐	☒	For null hypothesis testing, the test statistic (e.g. *F*, *t*, *r*) with confidence intervals, effect sizes, degrees of freedom and *P* value noted<br>*Give P values as exact values whenever suitable.*
☒	☐	For Bayesian analysis, information on the choice of priors and Markov chain Monte Carlo settings
☒	☐	For hierarchical and complex designs, identification of the appropriate level for tests and full reporting of outcomes
☐	☒	Estimates of effect sizes (e.g. Cohen's *d*, Pearson's *r*), indicating how they were calculated

Our web collection on statistics for biologists contains articles on many of the points above.

Software and code

Policy information about availability of computer code

Data collection	Flow cytometry: Flow cytometry data were collected using a BD Accuri™ C6 Plus and BD LSRFortessa™ (BD Biosciences).<br><br>Other: Qubit 4 Fluorometer; BioTek Synergy 4; Leica Aperio system; Spectral Ami Optical Imaging System.
Data analysis	Flow cytometry: Flow cytometry Standard (FCS) 3.0 files were analyzed with FlowJo v10 (FlowJo LLC).<br><br>Public data analyses: Glioblastoma (GBM) RNA-seq data (156 patients) was downloaded from TCGA. Correlation between normalized expression (FPKM) of selected genes was determined using corrplot R-package. Next, the GBM Patients (N = 156) were classified into high/low groups based on co-expression of PVR and NT5E using the upper and lower quartiles, which are 50% for each high and low groups. Normalized enrichment scores were calculated during a gene set enrichment test using fgsea v 3.11. All codes for bioinformatic analyses used are publicly available at https://github.com/natallah/GBM_TCGA_analyses.<br><br>Other: Microsoft Office 2007; Graphpad Prism 8; Aura; Aperio image analysis software;.

For manuscripts utilizing custom algorithms or software that are central to the research but not yet described in published literature, software must be made available to editors and reviewers. We strongly encourage code deposition in a community repository (e.g. GitHub). See the Nature Portfolio guidelines for submitting code & software for further information.

Data

Policy information about availability of data

All manuscripts must include a data availability statement. This statement should provide the following information, where applicable:

- Accession codes, unique identifiers, or web links for publicly available datasets
- A description of any restrictions on data availability
- For clinical datasets or third party data, please ensure that the statement adheres to our policy

The open access TCGA-GBM (RNA-seq) publicly available data was used in this study are available via Genomics Data Commons (GDC) data portal (https://portal.gdc.cancer.gov/projects/TCGA-GBM). This data was accessed via R-Bioconductor package TCGAbiolinks (accessed April 2020). Source data for all figures are provided with this paper and are available online. Source TCGA data for Figure 1A-C, S1 was downloaded from the Gene Expression Profiling Interactive Analysis (GEPIA2) database and is available online. The authors declare that all data supporting the findings of the study are available in the article, the Supplementary file, and the Source Data provided with this paper.

Research involving human participants, their data, or biological material

Policy information about studies with human participants or human data. See also policy information about sex, gender (identity/presentation), and sexual orientation and race, ethnicity and racism.

Reporting on sex and gender	In accordance with our IRB protocol approved by Purdue University's Institutional Review Board (IRB) (IRB-approved protocol #1804020540), samples were de-identifed and no sex or gender information was obtained.
Reporting on race, ethnicity, or other socially relevant groupings	In accordance with our IRB protocol approved by Purdue University's Institutional Review Board (IRB) (IRB-approved protocol #1804020540), samples were de-identifed and no race, ethnicity, or other socially relevant groups information was obtained.
Population characteristics	In accordance with our IRB protocol approved by Purdue University's Institutional Review Board (IRB) (IRB-approved protocol #1804020540), samples were de-identifed and population characteristics information was obtained.
Recruitment	Adult healthy donor were voluntarily recruited on Purdue University, West Lafayette campus. Due to population age characteristics of Purdue University (the majority betwwen age 18-30), potential age-related bias exists in the present study, but this information was not collected and likely has no impact on the outcome of this study.
Ethics oversight	Purdue University's Institutional Review Board (IRB) (IRB-approved protocol #1804020540)

Note that full information on the approval of the study protocol must also be provided in the manuscript.

Field-specific reporting

Please select the one below that is the best fit for your research. If you are not sure, read the appropriate sections before making your selection.

[x] Life sciences [ ] Behavioural & social sciences [ ] Ecological, evolutionary & environmental sciences

For a reference copy of the document with all sections, see nature.com/documents/nr-reporting-summary-flat.pdf

Life sciences study design

All studies must disclose on these points even when the disclosure is negative.

Sample size	Sample sizes were determined based on previously published studies from our laboratory (Wang et al., PNAS, 2021). These sample sizes are consistent with other reported therapeutics targeting GBM (Ma et al., Cancer Research, 2021; Han et al., Scientific Reports, 2015). Sample sizes are provided in the manuscript.
Data exclusions	No data were excluded from the analyses.
Replication	Experiments were repeated several times with independent samples, as indicated in the methods. Experiments were repeated at least three times with independent samples (such as independently differentiated NK cell cultures). All the repetitions of the experiments showed similar results or trend.
Randomization	For all studies throughout the manuscript, samples were randomly allocated between experimental groups. In all animal studies, animals were randomly allocated between treatment groups prior to treatment.
Blinding	The experiments were not performed in blind, as the same co-authors collected and analyzed the data obtain, due to personnel limitations.

Reporting for specific materials, systems and methods

We require information from authors about some types of materials, experimental systems and methods used in many studies. Here, indicate whether each material, system or method listed is relevant to your study. If you are not sure if a list item applies to your research, read the appropriate section before selecting a response.

Materials & experimental systems

n/a	Involved in the study
☐	☒ Antibodies
☐	☒ Eukaryotic cell lines
☒	☐ Palaeontology and archaeology
☐	☒ Animals and other organisms
☒	☐ Clinical data
☒	☐ Dual use research of concern
☒	☐ Plants

Methods

n/a	Involved in the study
☒	☐ ChIP-seq
☐	☒ Flow cytometry
☒	☐ MRI-based neuroimaging

Antibodies

Antibodies used

Antibodies used for flow cytometry and cell sorting were CD3-PeCy7 (BD, 563423, clone UCHT1), CD56-PeCy5.5 (Thermo, 35-0567-42, clone CMSSB), TIGIT-APC (Biolegend, 372705, clone A15153G), DNAM-1-BV510 (Biolegend, 338329, clone 11A8), Sytox Green (Thermo, S34860), Sytox Blue (Thermo, S34857), CD16-BUV395 (BD, 563785, clone 3G8), NKG2A-FITC (Miltenyi, 130-114-091, clone REA110), PD-1-BV510 (Biolegend, 329931, clone EH12.2H7), NKG2D-BV605 (Biolegend, 320831, clone 1D11), CD57-BV605 (Biolegend, 393303, clone QA17A04), CD69-BV650 (Biolegend, 310933, clone FN50), LAG-3-BV650 (Biolegend, 369315, clone 11C3C65), NKp30-BV711 (Biolegend, 325217, clone P30-15), TIM-3-BV711 (Biolegend, 345023, clone F38-2E2), CD94-PE (Biolegend, 305506, clone DX22), CD158b-APC/Fire750 (Biolegend, 312617, clone DX27), 41BB-APC/Fire750 (Biolegend, 309833, clone 4B4-1), CD96-PE (Biolegend, 338405, clone NK92.39), CD56-APC (Thermo, 17-0567-42, clone CMSSB), CD107a-PE (Biolegend, 328607, clone H4A3), IFNy-PerCPCy5.5 (Biolegend, 502525, clone 4S.B3), CD34-APC/Fire750 (Biolegend, 343535, clone 581), CD43-APC (Biolegend, 343205, clone CD43-10G7), CD45-BV711 (Biolegend, 304049, clone HI30), CD155-PE (Biolegend, 337609, clone SKII.4), CD73-PE (Biolegend, 344003, clone AD2), mCD155-PE (Biolegend, 132205, clone 4.24.1), mCD73-APC (Biolegend, 127209, clone TY/11.8), m4-1BB-APC (Biolegend, 106109, clone 17B5), mCD16/32-BV421 (Biolegend, 101331, clone 93), mNK1.1-BV510 (Biolegend, 108737, clone PK136), mCD3-BV711 (Biolegend, 100349, clone 145-2C11), mLAG-3-BV785 (Biolegend, 125219, clone C9B7W), mCD4-APC-Fire750 (Biolegend, 100459, clone GK1.5), mCD8a-PE-Dazzle594 (Biolegend, 100761, clone 53-6.7), mCD25-BV785 (Biolegend, 102051, clone PC61), mFoxP3-BV421 (Biolegend, 126419, clone MF-14), mPD-1-PE-Dazzle594 (Biolegend, 135227, clone 29F.1A12), mF4/80-BV510 (Biolegend, 123135, clone BM8), mMHC II-BV605 (Biolegend, 107613, clone M5/114.15.2), miNOS-PE (Biolegend, 696805, clone W16030C), mCD206-BV650 (Biolegend, 141723, clone C068C2), mArginase-1-APC (Thermo Fisher, 17-3697-82, clone A1exF5), mLy6G-PE-Cy7 (Biolegend, 127617, clone 1A8), GAL4 (Thermo, 33-8600), and Goat anti-mouse APC (Biolegend, 405308, clone Poly4053). Transcription factor staining was done using the Nuclear Transcription Factor staining kit (Biolegend, 424401). Tumor samples were dissociated using DNAse I and Collagenase IV purchased from Worthington Biochemical. Samples were run on a BD Fortessa and analyzed using FlowJo V10. Prior to use antibodies were validated for staining of primary NK cells or murine immune cells, respectively, and all antibodies were used at a 1:200 dilution.

Antibodies used in functional assays and in vivo assays were mouse anti-PVR (Leinco, C2833, clone 4.24.1), mouse anti-CD73 (BioXCell, BE0209, clone TY/23), anti-TIGIT (Biolegend, 613704, clone A15153A), and anti-CD73 (Thermo, 41-0200, clone 7G2). Human rhIL-15 was from Shenandoah Biotechnology. Dilutions used are described in the appropriate experimental methods sections.

For histological analysis following intracranial xenograft studies, whole brain samples were fixed, paraffin embedded, sectioned and stained for NKp46 (Abcam, ab224703, clone EPR22403-57), NKp46 (Abcam, ab283505, clone Mncr1.05), granzyme B (Abcam, ab4059), CD73 (Cell Signaling Technology, 13160, clone D7F9A), CD73 (Biolegend, 344002, clone AD2), CD155 (Cell Signaling Technology, 13544, clone D3G7H), CD3 (Abcam, ab11089, clone CD3-12), PD-1 (Abcam, ab214421, clone EPR20665), FoxP3 (Abcam, ab215206, clone EPR22102-37), and LAG-3 (Abcam, ab237720, clone CAL77).

Validation

CD3-PeCy7 (BD, 563423, clone UCHT1), and CD16-BUV395 (BD, 563785, clone 3G8) were validated by the manufacturer for human staining for flow cytometric use.

TIGIT-APC (Biolegend, 372705, clone A15153G), DNAM-1-BV510 (Biolegend, 338329, clone 11A8), PD-1-BV510 (Biolegend, 329931, clone EH12.2H7), NKG2D-BV605 (Biolegend, 320831, clone 1D11), CD57-BV605 (Biolegend, 393303, clone QA17A04), CD69-BV650 (Biolegend, 310933, clone FN50), LAG-3-BV650 (Biolegend, 369315, clone 11C3C65), NKp30-BV711 (Biolegend, 325217, clone P30-15), TIM-3-BV711 (Biolegend, 345023, clone F38-2E2), CD94-PE (Biolegend, 305506, clone DX22), CD158b-APC/Fire750 (Biolegend, 312617, clone DX27), 41BB-APC/Fire750 (Biolegend, 309833, clone 4B4-1), CD96-PE (Biolegend, 338405, clone NK92.39), CD56-APC (Thermo, 17-0567-42, clone CMSSB), CD107a-PE (Biolegend, 328607, clone H4A3), IFNy-PerCPCy5.5 (Biolegend, 502525, clone 4S.B3), CD34-APC/Fire750 (Biolegend, 343535, clone 581), CD43-APC (Biolegend, 343205, clone CD43-10G7), CD45-BV711 (Biolegend, 304049, clone HI30), CD155-PE (Biolegend, 337609, clone SKII.4), CD73-PE (Biolegend, 344003, clone AD2), mCD155-PE (Biolegend, 132205, clone 4.24.1), mCD73-APC (Biolegend, 127209, clone TY/11.8), m4-1BB-APC (Biolegend, 106109, clone 17B5), mCD16/32-BV421 (Biolegend, 101331, clone 93), mNK1.1-BV510 (Biolegend, 108737, clone PK136), mCD3-BV711 (Biolegend, 100349, clone 145-2C11), mLAG-3-BV785 (Biolegend, 125219, clone C9B7W), mCD4-APC-Fire750 (Biolegend, 100459, clone GK1.5), mCD8a-PE-Dazzle594 (Biolegend, 100761, clone 53-6.7), mCD25-BV785 (Biolegend, 102051, clone PC61), mFoxP3-BV421 (Biolegend, 126419, clone MF-14), mPD-1-PE-Dazzle594 (Biolegend, 135227, clone 29F.1A12), mF4/80-BV510 (Biolegend, 123135, clone BM8), mMHC II-BV605 (Biolegend, 107613, clone M5/114.15.2), miNOS-PE (Biolegend, 696805, clone W16030C), mCD206-BV650 (Biolegend, 141723, clone C068C2), mLy6G-PE-Cy7 (Biolegend, 127617, clone 1A8), and Goat anti-mouse APC (Biolegend, 405308, clone Poly4053) were validated by the manufacturer for the indicated species (human or mouse) for flow cytometric use.

CD56-PeCy5.5 (Thermo, 35-0567-42, clone CMSSB), Sytox Green (Thermo, S34860), Sytox Blue (Thermo, S34857), mArginase-1-APC (Thermo Fisher, 17-3697-82, clone A1exF5), and GAL4 (Thermo, 33-8600) were validated by the manufacturer for the indicated

species (human or mouse) for flow cytometric use.

NKG2A-FITC (Miltenyi, 130-114-091, clone REA110) was validated by the manufacturer for human staining for flow cytometric use.

Prior to use antibodies were validated for staining of primary NK cells or murine immune cells, respectively, and all antibodies were used at a 1:200 dilution.

Antibodies used in functional assays and in vivo assays were mouse anti-PVR (Leinco, C2833, clone 4.24.1), mouse anti-CD73 (BioXCell, BE0209, clone TY/23), anti-TIGIT (Biolegend, 613704, clone A15153A), and anti-CD73 (Thermo, 41-0200, clone 7G2), and were validated at the indicated concentrations by the manufacturer for the indicated species (human or mouse) for functional blockade use

Eukaryotic cell lines

Policy information about cell lines and Sex and Gender in Research

Cell line source(s)	GL261 (PTA-5893), HCN-2 (CRL-3592), U87-MG (HTB-14) and K562 (CLL-24) cells were purchased from ATCC. SJ-GBM2 cells were provided by Dr. Karen Pollok, Indiana University School of Medicine, and were originally obtained from the Children's Oncology Group and were obtained from a female patient. GBM43 and GBM10 cells were provided by Dr. Karen Pollok, Indiana University School of Medicine. GBM43 and GBM10 xenograft tissues from male patientswere provided by Dr. Jann Sarkaria (Mayo Clinic, Rochester MN), and cell lines were subsequently derived from xenograft tissues in Dr. Pollok's lab. The hiPSC01 iPS cell line was generated by us at the Purdue Institute for Integrative Neuroscience Cell Engineering core facility. Healthy male dermal fibroblasts (ReprocellUSA) were reprogrammed using the StemRNA 3rd Generation Reprogramming kit (Reprocell USA) according to the manufacturer's protocol.
Authentication	GL261, HCN-2, U87-MG, and K562 cell identity was confirmed by morphology. SJ-GBM2, GBM10, and GBM43 cell identity was confirmed by DNA fingerprint analysis (IDEXX BioResearch) for species and baseline short-tandem repeat analysis testing and were found to be entirely human. hiPSC01 cell identy was confirmed by STR analysis.
Mycoplasma contamination	All cell lines are mycoplasma negative.
Commonly misidentified lines (See ICLAC register)	No misidentified line included.

Animals and other research organisms

Policy information about studies involving animals; ARRIVE guidelines recommended for reporting animal research, and Sex and Gender in Research

Laboratory animals	Male and female 6- to 8-week-old C57BL/6 (Charles River), NOD.Cg-Prkdcscid IL2rgtm1Wji/SzJ (NSG; Jackson Lab) and NOD.Cg-Prkdcem26Cd52 IL-2em26Cd22/NjuCrl (NCG; Charles River) mice were maintained by our lab or at the Purdue Center for Cancer Research. All animal experiments described in this study were approved by the Purdue University Animal Care and Use Committee. Mice were housed in a 12 hour/12hour light/dark cycle with a temperature of 18-23C and relative humidity of 40-60%.
Wild animals	No wild animals involved.
Reporting on sex	Male and female mice were included in these studies. No significant differences between sex were observed.
Field-collected samples	No field-collected samples involved.
Ethics oversight	All experiments were conducted in compliance with all relevant ethical regulations and standards. All animal experiments described in this study, utilizing C57BL/6, NOD.Cg-Prkdcscid IL2rgtm1Wji/SzJ (NSG), and NOD.Cg-Prkdcem26Cd52 IL-2em26Cd22/NjuCrl (NCG) mice, were approved by the Purdue University Animal Care and Use Committee.

Note that full information on the approval of the study protocol must also be provided in the manuscript.

Plants

Seed stocks	N/A
Novel plant genotypes	N/A
Authentication	N/A

Flow Cytometry

Plots

Confirm that:

- [x] The axis labels state the marker and fluorochrome used (e.g. CD4-FITC).
- [x] The axis scales are clearly visible. Include numbers along axes only for bottom left plot of group (a 'group' is an analysis of identical markers).
- [x] All plots are contour plots with outliers or pseudocolor plots.
- [x] A numerical value for number of cells or percentage (with statistics) is provided.

Methodology

Sample preparation	Cells were harvested directly. The staining was processed in the dark with the amount of corresponding antibodies for a certain time. After that, the cells were collected and washed with FACS buffer, and re-suspended with the specific buffer for FACS analysis.
Instrument	BD Accuri™ C6 Plus; BD LSRFortessa™.
Software	Flow Jo v10.
Cell population abundance	Sorting was not used in this study.
Gating strategy	Generally, cells were firstly gated using forward forward scatter area (FSC-A) versus scatter area (SSC-A) for selection of target cells as well as to remove some dead cells or cell debris, then forward scatter area (FSC-A) versus forward scatter height (FSC-H) for double exclusion. The dead cells were further excluded by live/dead staining. Then the target markers' expression was analyzed by the settled channel. The unstained cells, cells stained with isotype antibodies or FMO staining were settled as the appropriated controls. For the experiments involving co-cultured cells (NK cells together with target cells including cancer cells or normal cells), the NK cells were stained CD56+CD3- to distinguish NK cells from target cells.

- [x] Tick this box to confirm that a figure exemplifying the gating strategy is provided in the Supplementary Information.
