# Supplementary material for: synNotch-programmed iPSC-derived NK cells usurp TIGIT and CD73 activities for glioblastoma therapy
Source: Nat Commun. 2024 Mar 1;15:1909. doi: 10.1038/s41467-024-46343-3 (PMC10907695; doi:10.1038/s41467-024-46343-3)
Supplement: Supplementary file 4 — Description of Additional Supplementary Files [file 41467_2024_46343_MOESM4_ESM.pdf]

Description of Additional Supplementary Files

1. **Supplementary Data 1.** List of upregulated DE genes between $CD155^{high}/CD73^{high}$ and $CD155^{low}/CD73^{low}$ GBM patients

2. **Supplementary Data 2.** List of downregulated DE genes $CD155^{high}/CD73^{high}$ and $CD155^{low}/CD73^{low}$ GBM patients
